# Supplementary figures and images for: Incretin-Based Multi-Agonist Peptides Are Neuroprotective and Anti-Inflammatory in Cellular Models of Neurodegeneration
Source: Biomolecules. 2024 Jul 19;14(7):872. doi: 10.3390/biom14070872 (PMC11275108; doi:10.3390/biom14070872)

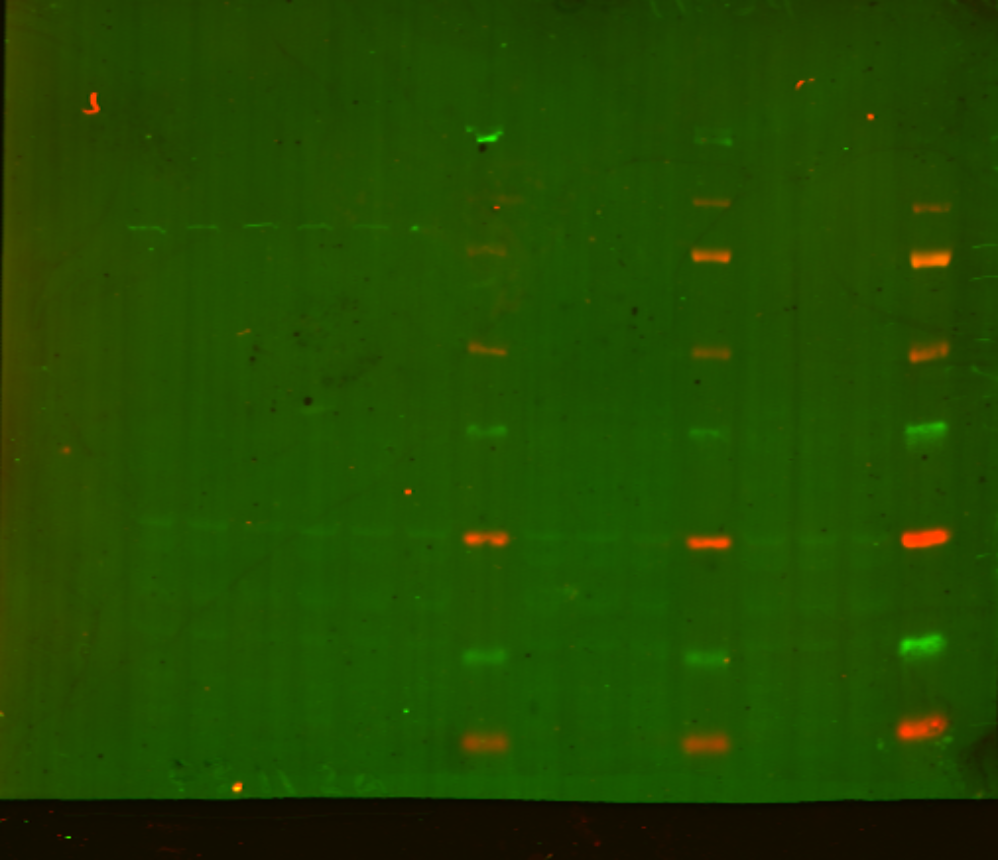

Supplement: Supplementary file 1 [file biomolecules-14-00872-s001.zip › 20240312 iNOS Western low res.tif]

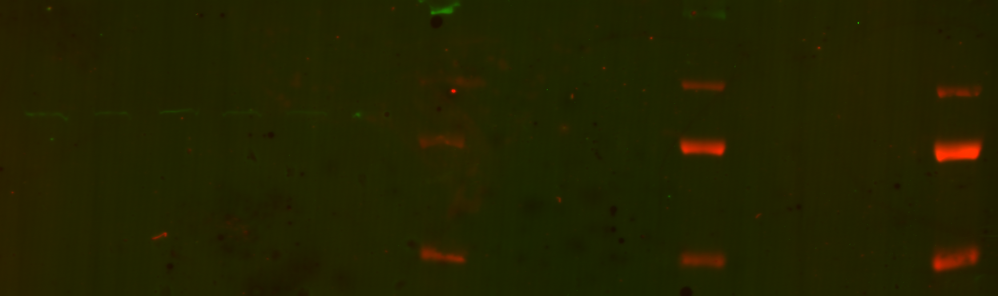

Supplement: Supplementary file 1 [file biomolecules-14-00872-s001.zip › 20240312 iNOS Western zoomed in.tif]

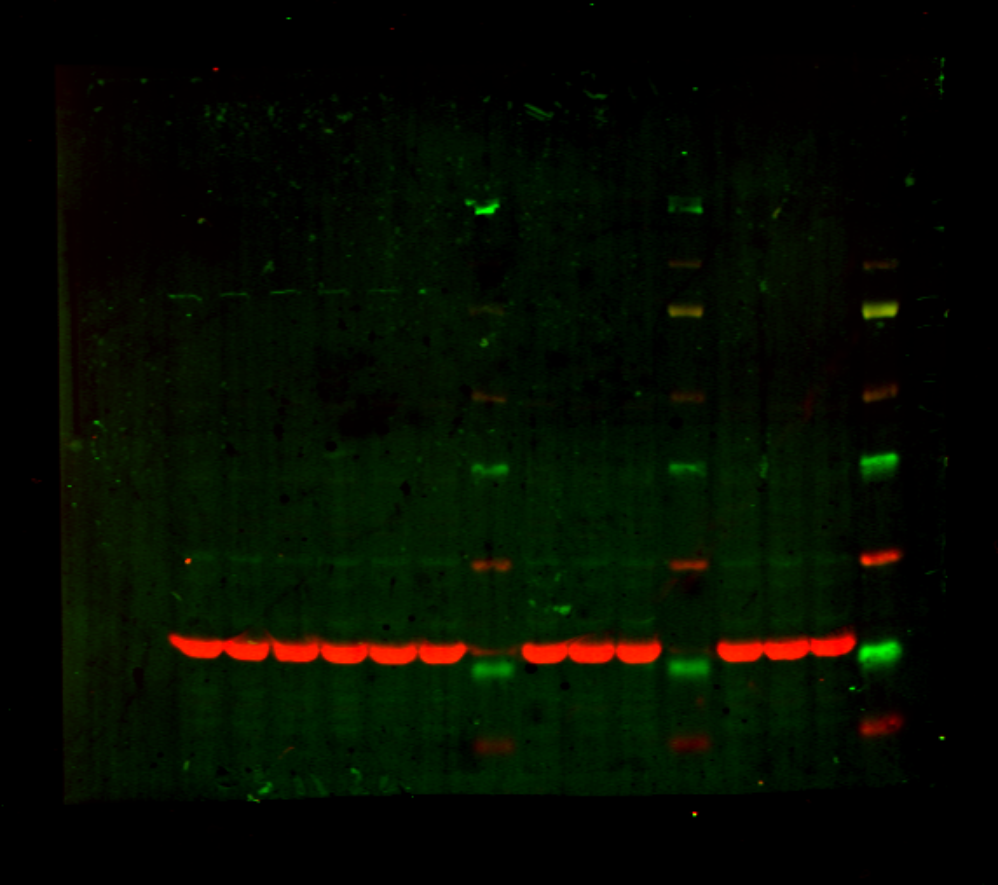

Supplement: Supplementary file 1 [file biomolecules-14-00872-s001.zip › 20240313 GAPDH western whole membrane.tif]

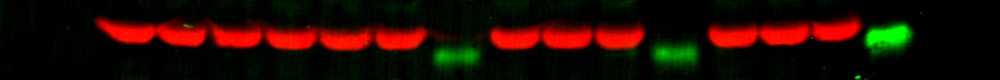

Supplement: Supplementary file 1 [file biomolecules-14-00872-s001.zip › 20240313 GAPDH western zoomed in.tif]

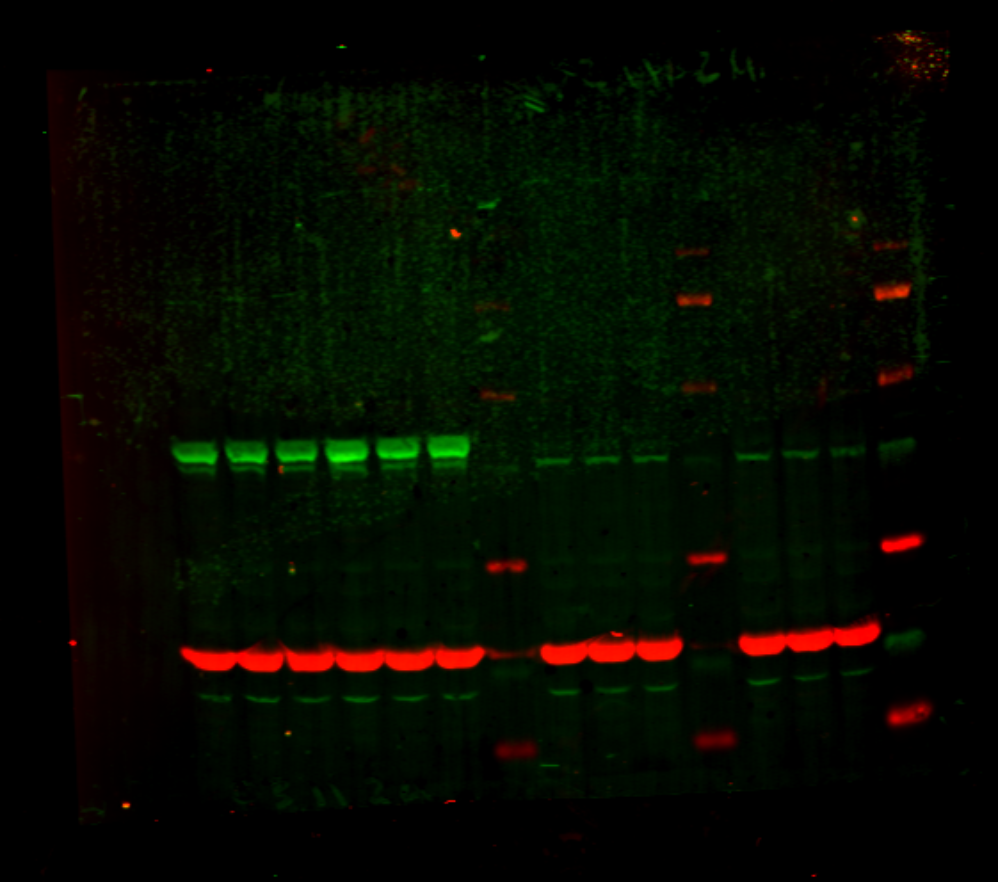

Supplement: Supplementary file 1 [file biomolecules-14-00872-s001.zip › 20240314 COX2 whole membrane.tif]

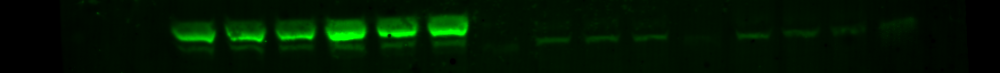

Supplement: Supplementary file 1 [file biomolecules-14-00872-s001.zip › 20240314 COX2 zoomed in.tif]

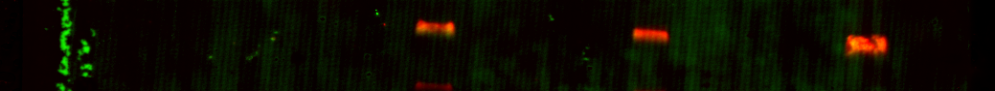

Supplement: Supplementary file 1 [file biomolecules-14-00872-s001.zip › 20240320 iNOS high res control.tif]

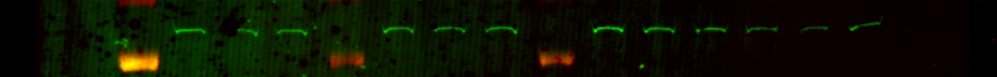

Supplement: Supplementary file 1 [file biomolecules-14-00872-s001.zip › 20240320 iNOS high res lps.tif]

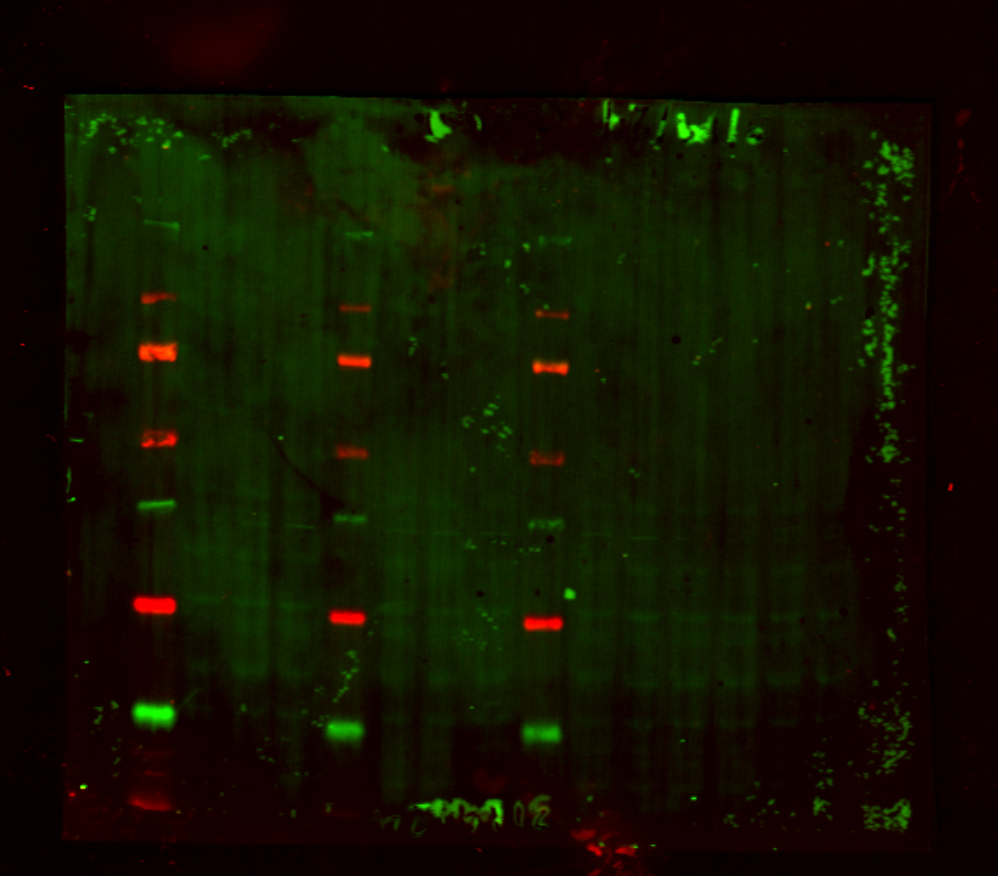

Supplement: Supplementary file 1 [file biomolecules-14-00872-s001.zip › 20240320 iNOS whole membrane control.tif]

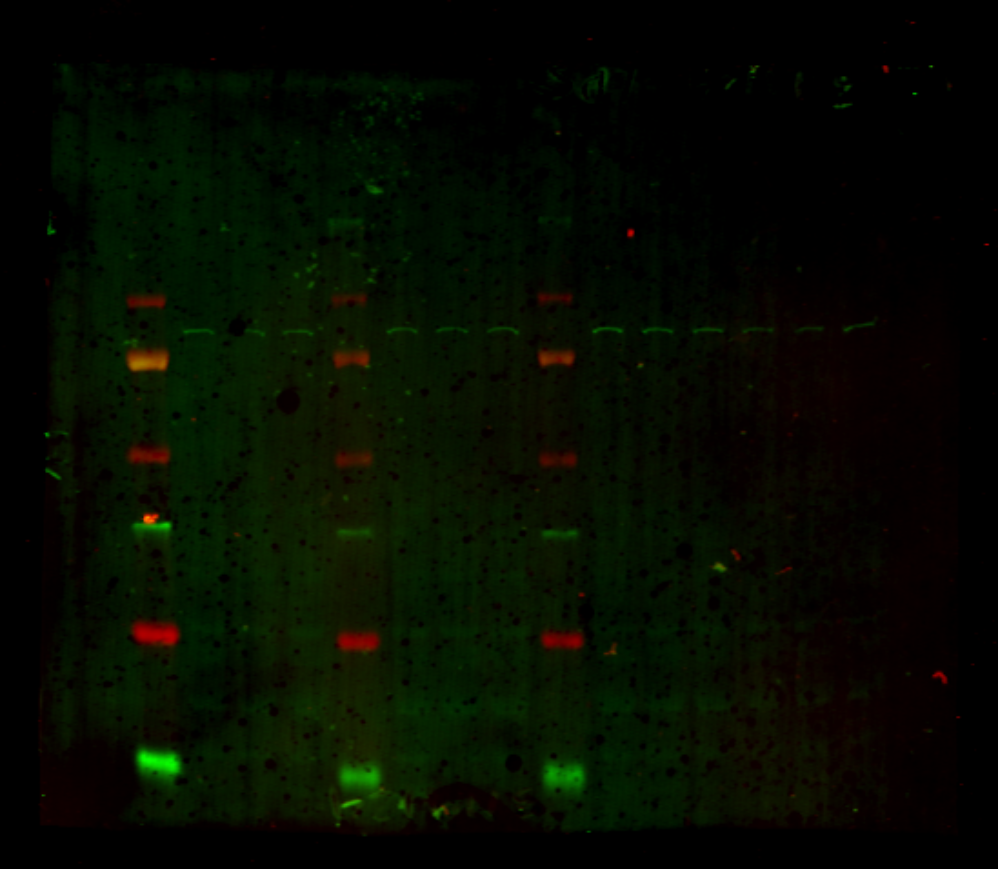

Supplement: Supplementary file 1 [file biomolecules-14-00872-s001.zip › 20240320 iNOS whole membrane lps.tif]

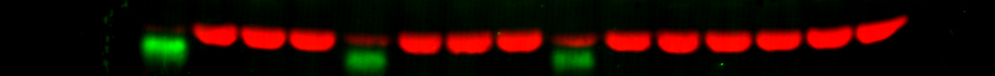

Supplement: Supplementary file 1 [file biomolecules-14-00872-s001.zip › 20240321 GAPDH high res control.tif]

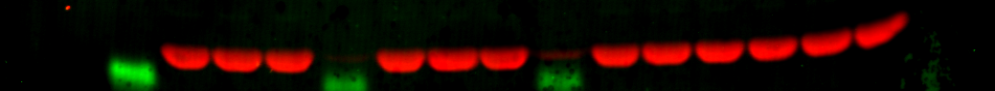

Supplement: Supplementary file 1 [file biomolecules-14-00872-s001.zip › 20240321 GAPDH high res lps.tif]

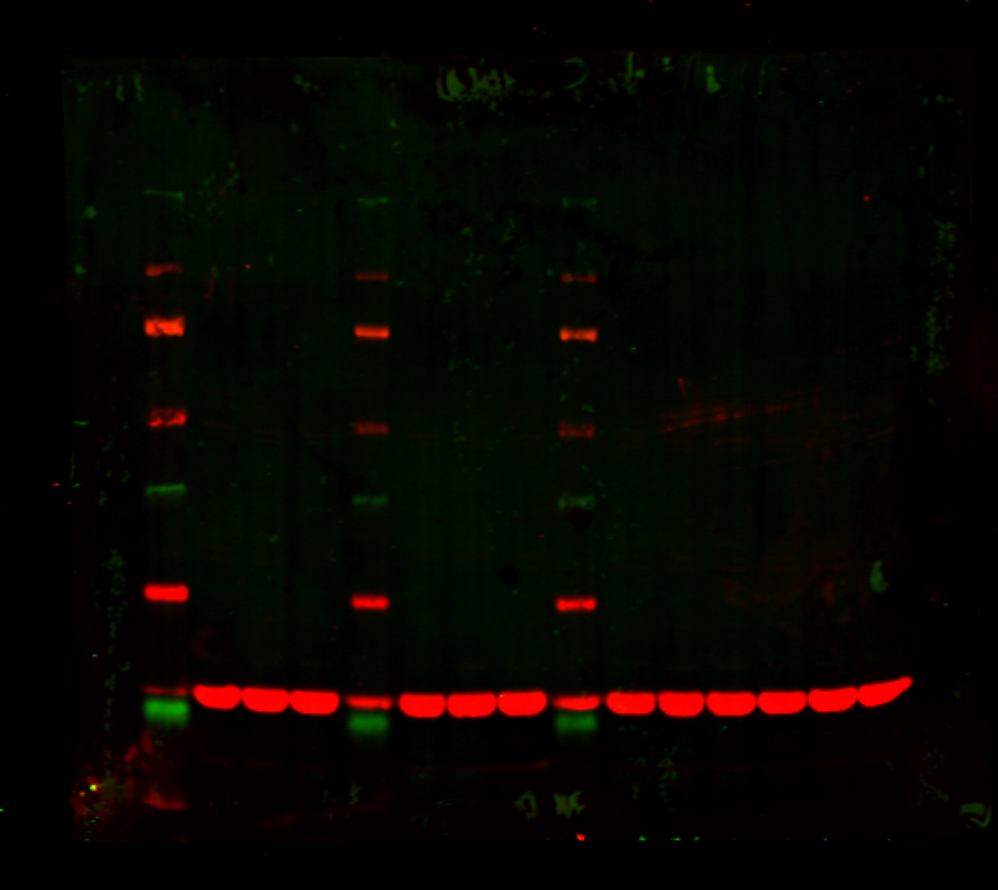

Supplement: Supplementary file 1 [file biomolecules-14-00872-s001.zip › 20240321 GAPDH whole membrane control.tif]

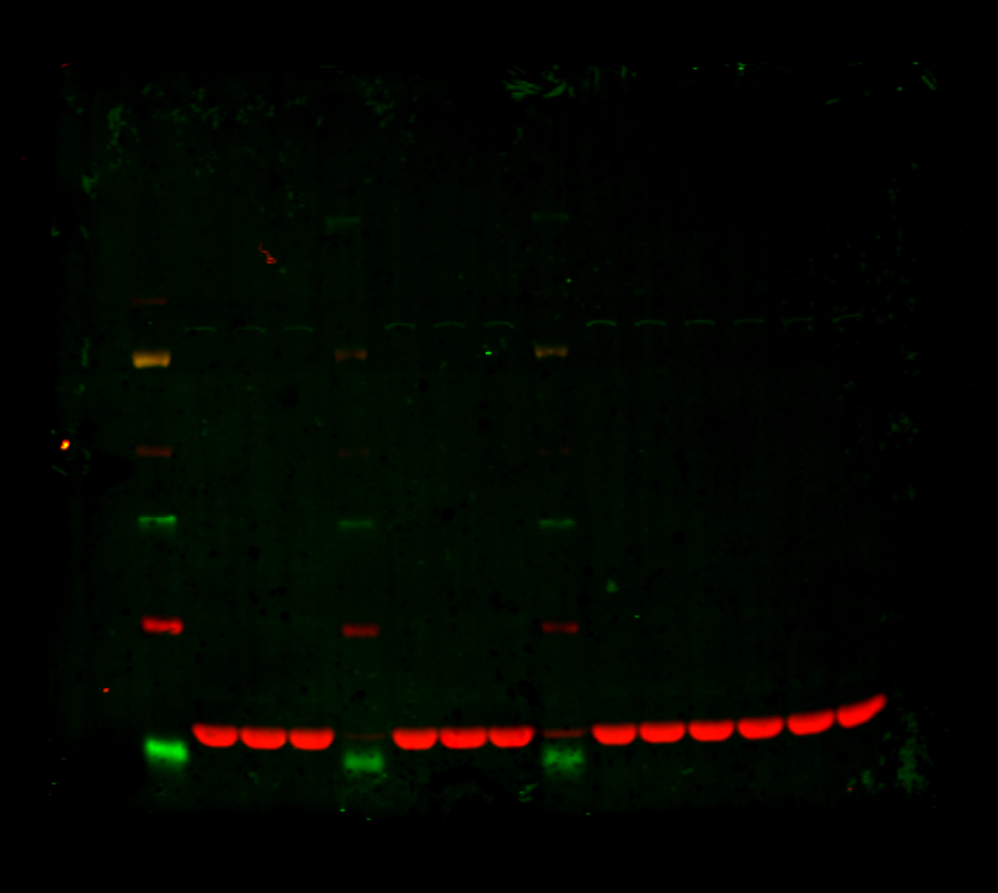

Supplement: Supplementary file 1 [file biomolecules-14-00872-s001.zip › 20240321 GAPDH whole membrane lps.tif]

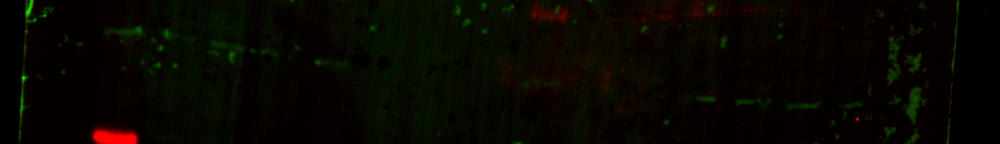

Supplement: Supplementary file 1 [file biomolecules-14-00872-s001.zip › 20240322 COX2 high res control.tif]

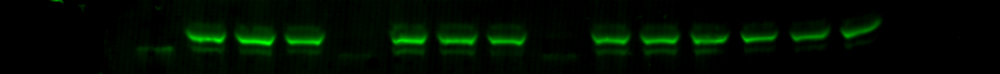

Supplement: Supplementary file 1 [file biomolecules-14-00872-s001.zip › 20240322 COX2 high res lps.tif]

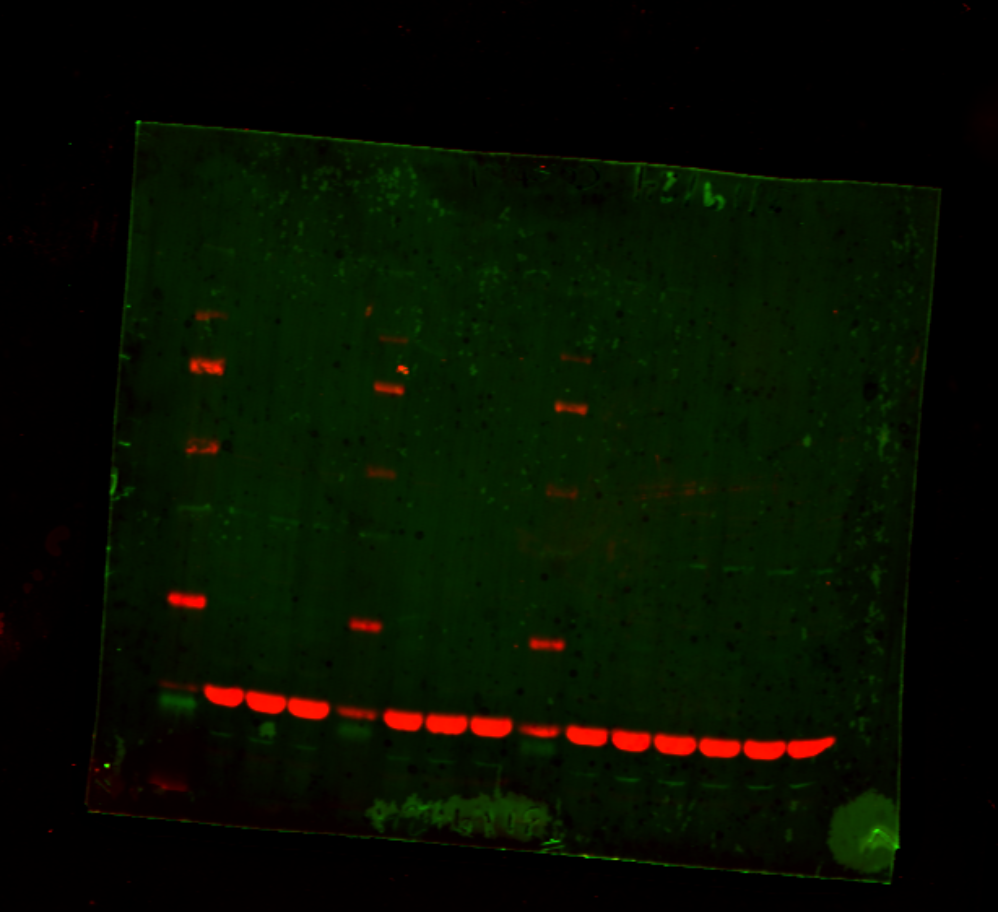

Supplement: Supplementary file 1 [file biomolecules-14-00872-s001.zip › 20240322 COX2 whole membrane control.tif]

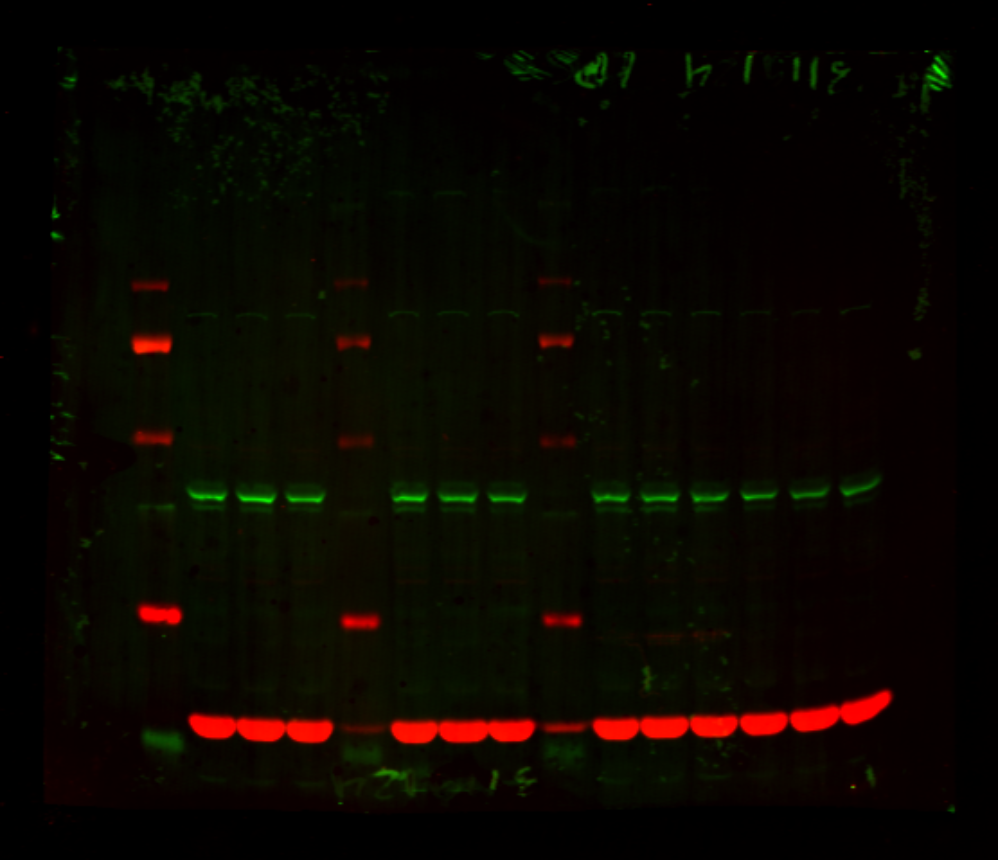

Supplement: Supplementary file 1 [file biomolecules-14-00872-s001.zip › 20240322 COX2 whole membrane lps.tif]
